# How does family functioning effect the outcome of family based treatment for adolescents with severe anorexia nervosa?

**DOI:** 10.1186/s40337-017-0184-9

**Published:** 2017-12-13

**Authors:** Andrew Wallis, Jane Miskovic-Wheatley, Sloane Madden, Paul Rhodes, Ross D. Crosby, Li Cao, Stephen Touyz

**Affiliations:** 1 Eating Disorder Service, The Sydney Children’s Hospital Network, Westmead Campus, Sydney, Australia; 2 School of Psychology, The University of Sydney, Sydney, Australia; 3 Neuropsychiatric Research Institute, Fargo, ND USA; 4 University of North Dakota School of Medicine and Health Sciences, Fargo, ND USA; 5 Department of Adolescent Medicine, The Sydney Children’s Hospital Network, Westmead Campus, Locked Bag 4001, Westmead, NSW 2145 Australia

**Keywords:** Anorexia nervosa, Adolescents, Family functioning, Attachment, Family based treatment

## Abstract

**Background:**

The aim of this research was to investigate the relationship between family functioning, adolescent-parent attachment and remission, as well as changes in these variables over time for adolescents with severe anorexia nervosa treated with family based treatment (FBT). Understanding how families respond to treatment is important because the family will be the ongoing context for psychosocial development in the longer term. The relationship between family functioning and outcome is also an important variable because it is potentially modifiable during treatment and this may improve outcome.

**Methods:**

Fifty-seven female adolescents treated with FBT in a randomised controlled trial were assessed at baseline, FBT session 20 and 12-months post FBT session 20. Data on family functioning and adolescent-parent attachment was collected from patients and their parents at each time point. A series of regression analyses were used to determine the relationship between family functioning and comorbidity at baseline, and the relationship with remission status over time. Repeat measure mixed-effects models were used to assess changes in family functioning and attachment quality over time.

**Results:**

Greater adolescent perceived family functioning impairment was positively related to psychiatric comorbidity at the start of treatment. Conversely, better family functioning predicted higher self-esteem and stronger attachment quality. Adolescent’s reporting better general family functioning, communication and problem solving were more likely to be remitted at session 20, but not at 12-month follow-up. There was no overall improvement in family functioning for any respondent either during treatment or at follow-up, and no significant relationship between change and remission at either session 20 or follow-up.

**Conclusions:**

The adolescent’s perspective on family functioning at the start of treatment impacts on a positive outcome. Addressing family issues earlier in FBT may be important for some patients. Further research is needed in this area to determine how these findings could be integrated into the current FBT model.

**Trial registration:**

Australian Clinical Trials Register number: ACTRN012607000009415 (www.anzctr.org.au).

## Plain English Summary

In this study, the adolescent’s view of family functioning was critical. Adolescents with more positive views had lower mental health difficulties, better self-esteem and better relationship quality with their parents. More positive adolescent but not parent views were related to better outcome at the end of treatment. There were no positive changes in family functioning during or after FBT with fathers, indicating increased difficulty during treatment that resolved after treatment. Addressing family issues for adolescents earlier in treatment is important and may improve outcome. Further research is needed to determine how these finding could be integrated into the current family based treatment model.

## Background

Family therapy approaches (FT) for adolescent anorexia nervosa (AN) are currently recommended as first line outpatient treatment [1–4]. A number of family therapy approaches specific to AN have developed in the UK and USA since the mid 1980’s that share a common underlying tenet that the family is a key treatment resource, and the reduction of AN symptoms to reverse starvation should be the initial focus of treatment. While originally developed and tested at the Maudsley Hospital in the UK, dissemination has been aided by the availability of the Maudsley Service treatment manual, and Family Based Treatment manual (FBT) in the USA [5, 6]. The evidence for FT continues to expand with randomised controlled trials (RCT) indicating that approximately 25-50% of patients achieve changes in weight and eating disorder symptoms indicative of remission at the end of treatment and significant weight improvement occurs for a substantial majority of patients [7–14].

While FT is efficacious, limited information exists to help improve treatment outcomes when response is poor. Thus, understanding more about moderators and mediators of outcome remains an important goal to improve treatment response [15]. Much of what we know about poor treatment response for adolescents identifies pre-existing factors such as length of illness, prior hospitalisation and being older, which are not modifiable when treatment begins [13, 16]. However, family functioning is a variable that can be modified during treatment, potentially improving treatment response [17]. While family factors are not recognised as causing AN [18], the illness does impact on the family and thus family functioning can operate as a maintaining factor effecting treatment response [19, 20].

A number of elements of family functioning have been related to outcome in previous FT studies. Positive relationship patterns at the start of treatment, such as parental warmth (i.e. emotional attunement) and family cohesion have been associated with good end of treatment outcome [21, 22]. Similarly, better family maintenance (i.e. organisation and control) early in treatment predicts good outcome [22]. Negative impacts on outcome have been seen with a communication style characterised by critical comments from mother to child [8, 13, 17].

Positive changes in family functioning appear to improve outcome in FT. A recent study of FBT and individual therapy [23, 24] identified a number of positive changes in family functioning during treatment that were predictive of end of treatment remission status. Specifically, these included; mother-reported general family functioning (i.e. overall health), father-reported problem solving (i.e. capacity to problem solve critical issues that threaten the families integrity), and adolescent-reported roles (i.e. family members knowing what, and when they need to take on tasks). Similarly, an earlier study identified improvement in family cohesion and emotional expressiveness 6 months into treatment predicted better outcome at the end of treatment [22]. While not the primary aim of the FT models referred to here, changes in family functioning and relationships during treatment are likely given the focus on problem solving, role clarity and direct communication in the early phases of treatment and the focus on healthy adolescent-parent relationship patterns later in treatment [5]. Although, the reverse may occur if treatment progress is poor [25].

The relationship between pre-treatment family functioning, changes in family functioning and remission for adolescent’s with higher levels of AN symptomology is unclear. Previous studies have indicated that patients with higher levels of eating disorder psychopathology and comorbid psychiatric symptomatology tend to remit at lower rates [16, 22]. Poorer general family functioning is also related to greater levels of eating disorder pathology and psychiatric symptoms [24]. Family functioning may be more critical to outcome for these patients [16] because mobilising the family in the face of elevated eating disorder, mood and anxiety symptoms may be too challenging. In addition, families in this situation may need to be more actively engaged in managing and supporting their offspring over a longer period, placing additional strain on family wellbeing, and adolescent-parent attachment, which could lead to a deterioration in function over time [26, 27].

Whether changes in family functioning or adolescent-parent attachment occur, or predict remission status in the longer term in adolescents with severe AN is unclear. Previous FBT studies investigating the link between family functioning and treatment response have primarily involved individuals with low levels of AN psychopathology, psychiatric comorbidity and medical complications [24]. Given hospitalisation is also a predictor of poor outcome [13, 16, 28], patients requiring medical admission, combined with these other markers of severity, may be at increased risk of poor response and are an important population to examine.

This post hoc exploratory study investigated the impact of family functioning and adolescent-parent attachment on treatment outcomes for adolescents with high levels of AN psychopathology, hospitalised for management of medical instability prior to manualised Family Based Treatment (FBT) as described by Lock and Le Grange [5]. The first aim was to investigate the salience of the relationship between adolescent-, mother- and father-reported general family functioning health and eating disorder psychopathology, psychiatric co-morbidity and adolescent-parent attachment at the beginning of treatment, in this unwell patient group. The second aim was to investigate whether mother-, father- and adolescent-reported family functioning, measured on multiple dimensions, adolescent-parent attachment quality at the beginning of treatment, and changes in family functioning and adolescent-parent attachment quality during treatment, predicted remission at FBT session 20 and at 12-month follow-up. The third aim was to examine changes in family functioning dimensions and parent-adolescent attachment quality over the course of treatment and follow-up for mothers, fathers and adolescents.

## Method

### Participants

Participants (*n* = 57) were a subset from a previously reported randomised controlled trial investigating the role of inpatient weight restoration prior to outpatient family-based treatment [12]. To be eligible for inclusion in the original study, participants met DSM-IV criteria for AN of less than 3 years duration [29] and were medically unstable at the time of hospital admission [hypothermic (temperature < 35.5 °C), brady-cardic (heart rate < 50 beats/min), hypotensive (blood pressure < 80 mmHg systolic and <40 mmHg diastolic), orthostatic instability (pulse increase >20 beats/min, systolic blood pressure decrease >20 mmHg) or QT interval corrected for heart rate > 0.45 s]. No participants were excluded due to co-morbid psychiatric conditions, no participants had been hospitalised for AN previously or had received FBT before the RCT. Eligible participants were randomly assigned to either medical stabilisation (MS) or minimum weight restoration (WR) before outpatient FBT. Further details have been reported in the main trial paper [12]. Participants included in the current study were patients from either group (MS = 28; WR = 29) who completed the treatment protocol (i.e. inpatient admission and 20 sessions of FBT, unless treatment goals were met prior) and reliably completed the assessment measures of interest at session 20 and 12-month follow-up. This subset represented 82.6% of the 69 patients who completed the RCT protocol. There were no significant differences between this subset (*n* = 57) and the RCT cohort on any baseline variable, and no significant differences between the MS and WR patients in this study for age, illness duration, weight or eating disorder psychopathology at baseline. The only significant difference between the MS and WR group was weight at discharge, i.e. start of FBT, as determined by the RCT protocol. There was no difference in remission rate between MS and WR patients at any assessment point in this study (*n* = 57). Therefore, the MS and WR groups were combined for this analysis.

### Intervention

FBT [5] is a three-phase model that directs parents to initially take responsibility for weight gain and a return to normal eating, so as to reverse the starvation caused by AN. As treatment progresses into phases two and three, responsibility for eating is gradually handed back to the adolescent so they can progress independently with both food and normal adolescent tasks. This progression aims to ensure safety, but is also developmentally sensitive, shifting the focus back to the adolescent when they are able to manage the AN [5, 30]. FBT therapists were three psychologists and a social worker. Supervision was provided weekly by experienced supervisors (AW and PR) with more that 5 years experience with FBT. Treatment fidelity was assessed by reviewing videos of a random sample (5%) of sessions by an author of the treatment manual [12].

### Assessments

Participants were assessed at baseline, session 20 of FBT and 12-months after session 20. Remission outcome was defined as percent Expected Body Weight (%EBW) ≥ 95%, calculated using the Centre for Disease Control (CDC) growth charts [31] and an Eating Disorder Examination (EDE) Global Score within 1 SD of community norms [32, 33]. Previous FBT studies have utilised this definition [23, 34]. Family functioning and adolescent-parent attachment quality outcomes were assessed with the Family Assessment Device and Inventory of Parent and Peer Attachment as described below.

*Baseline clinical characteristics* included; age, illness duration (months), AN subtype [AN restricting (AN-R); AN binge/purging (AN-BP)], %EBW and Eating Disorder psychopathology, assessed with the Eating Disorder Examination (EDE) Global Score. The EDE is a structured and validated clinical interview [32, 33].

*Co-morbid psychiatric clinical characteristics* included depression, anxiety and obsessive compulsive disorder, assessed with the Schedule for Affective Disorders and Schizophrenia for School Aged Children (KSADS; [35]), with both patient and parent(s) interviewed. Adolescents reported depression, anxiety and Obsessive Compulsive Disorder (OCD) symptoms (RCADS; [36]), OCD impact (ChOCI-R; [37]), self-esteem [Rosenberg Self Esteem Scale (RSES; [38]) and global health functioning as assessed with the Child Health Questionnaire (CHQ-CF87; [39]).

*Family functioning* was measured using the Family Assessment Device (FAD; [40]) from adolescent, mother and father perspectives, or by one parent in non-intact families. The FAD is a self-report measure with 60-items divided into seven subscales assessing aspects of functioning and relationships within the family based on the McMaster model of family functioning [41]. The subscales are: Problem solving (ability to solve problems that affect the integrity and function of the family), Communication (clarity and directness of verbal messages to intended family member), Roles (Ability to define, establish and assign functions), Affective responsiveness (Openness to experience and show affect appropriate to situations, including emergencies), Affective involvement (interest and value placed on each other’s concerns and activities), Behaviour control (expression, maintenance and patterns of behaviour standards), and a unique General Functioning scale assessing the overall health of the family. The FAD is scored on a Likert scale 1 (strongly agree) to 4 (strongly disagree). Higher scores indicate greater difficulty, with General Functioning scale scores greater than 2.0 indicating clinical impairment [42]. The FAD is well-validated and reliable with internal consistency for the subscales between.72-.92 [42]. Internal consistencies for all respondent subscale scores were high with 87 fathers, 90 for mothers, and 90 for adolescents. The FAD been used in previous eating disorder studies and is completed by adolescent, mother and father [24, 43, 44].

*Adolescent attachment relationship quality with parents* was assessed with the Inventory of Parent and Peer Attachment (IPPA-45; [45, 46]). The IPPA is a 45-item questionnaire that aims to tap into the affective cognitive expectancies associated with representations of the quality, rather than categorisation of the attachment relationships between a young person and their mother and father. The IPPA is well validated and reliable [46] and has used in previous eating disorder studies. The IPPA-45 has reported internal consistencies of alpha 85 -.92 [47]. The adolescent completes the IPPA and only the mother and father scales were used in this study.

### Statistical analysis

Distributional characteristics of outcome variables were evaluated using graphical displays and tests of distributional statistics (i.e., skew, kurtosis) to identify deviations from normality. To investigate the first aim, a series of regression analyses were used to examine the relationship between baseline variables and general family functioning for mothers, fathers and adolescents. FAD General Functioning subscale (GF) scores for mothers, fathers and adolescents were included simultaneously as predictors of baseline variables. To investigate the second aim, logistic regression analyses were used to evaluate the relationship between baseline, and changes, in family functioning using all FAD subscales and adolescent-parent attachment quality to remission at session 20 and 12-month follow-up. Change in FAD subscales and attachment were used as the independent variables and baseline scores as the covariate. Finally, to investigate the third aim, change in family functioning and adolescent-parent attachment quality between baseline, session 20 and 12-month follow-up, repeated measures mixed-effects models were used for mother, father and adolescent, on each FAD subscale, and attachment to mother and attachment to father on the IPPA. All analyses was done using available data for each mother, father and adolescent at each time point, with an alpha level of .05 for all statistical tests. Data were analysed using SPSS Version 21 for Windows.

## Results

### Patient characteristics

Adolescents had a mean age of 14.72 years (*SD* = 1.39) with duration of illness of 7.49 months (*SD* = 6.31). Sixty five percent had restricting AN (AN-R) and 35% had AN binge-purge sub-type (AN-BP). Expected Body weight (EBW) on admission was 78.30% (*SD* = 6.14). The group had high levels of eating disorder psychopathology with Global EDE mean of 3.16 (*SD* = 1.18), and high levels of comorbid psychiatric clinical illness: anxiety (27 participants, 47%); OCD (12 participants, 21%); and depression (30 participants, 53%). All adolescents were medically unstable at baseline requiring hospitalisation prior to family-based treatment. Most parents were married, with 11 (19.3%) either divorced or separated, including one reconstituted family. Two families were widowed.

Mean %EBW at session 20 was 94.26 (7.80) and 95.50 (9.87) at 12-month follow-up, with remission rate of 19% (11 patients) at session 20 and 32% (18 patients) at 12-month follow-up. Table 1 describes FAD and IPPA means at each time point. Higher FAD scores indicate poorer functioning and higher IPPA scores better attachment quality. FAD subscale means above established clinical impairment cut-offs [42] are noted in the table. Most FAD means were below the established clinical impairment cut-offs. At baseline adolescents were above the cut-off for Behaviour Control and parents below on all subscales. At session 20 adolescents and fathers were above the cut-off for communication and affective involvement, and fathers for General Functioning (GF). At 12-month follow-up adolescents and fathers were above the cut-off for Affective Involvement.

**Table 1:** FAD and IPPA at baseline, session 20 and 12 month follow up

	Adolescent *n* = *57* Mean (SD)			Mother *n* = *55* Mean (SD)			Father *n* = *46* Mean (SD)		
Family Assessment Device	Baseline	S20	12mFU	Baseline	S20	12mFU	Baseline	S20	12mFU
General Functioning	1.93 (.59)	1.87 (.58)	1.89 (.50)	1.91 (.46)	1.84 (.46)	1.83 (.41)	1.90 (.39)	2.06^a^ (.40)	1.95 (.35)
Problem Solving	2.07 (.53)	2.00 (.44)	2.03 (.50)	2.01 (.42)	1.99 (.38)	1.98 (.38)	2.01 (.30)	2.09 (.28)	2.01 (.31)
Communication	2.15 (.46)	2.20^a^ (.46)	2.18 (.42)	1.99 (.36)	2.02 (.41)	2.05 (.37)	2.04 (.29)	2.20^a^ (.32)	2.08 (.33)
Roles	2.15 (.47)	2.04 (.41)	2.02 (.43)	2.13 (.30)	2.16 (.32)	2.10 (.36)	2.17 (.43)	2.24 (.32)	2.15 (.40)
Affective Responses	2.08 (.66)	1.99 (.54)	2.00 (.56)	1.90 (.63)	1.92 (.53)	1.92 (.49)	2.01 (.47)	2.16 (.45)	2.12 (.42)
Affective Involvement	2.06 (.52)	2.11^a^ (.51)	2.14^a^ (.55)	1.98 (.40)	2.00 (.46)	1.97 (.37)	2.01 (.40)	2.10^a^ (.47)	2.10^a^ (.45)
Behaviour Control	2.02^a^ (.45)	1.88 (.47)	1.88 (.44)	1.83 (.42)	1.79 (.44)	1.69 (.38)	1.78 (.39)	1.81 (.31)	1.80 (.44)
Inventory of Parent and Peer Attachment									
Mother Attachment	54.29 (9.44)	51.59 (6.66)	51.98 (6.78)						
Father Attachment	51.33 (10.38)	48.55 (6.66)	48.83 (8.67)						

^a^Mean above the impaired functioning cutoffs established by Miller, et al. 1985 [42]

S20 = Session 20; 12mFU = 12 month follow up; Cutoffs - General Functioning ≥2.0; Problem Solving ≥2.2; Communication ≥2.2; Roles ≥2.3; Affective Responsiveness ≥2.2; Affective Involvement ≥2.1; Behaviour Control ≥1.9

### Relationship between general family functioning and baseline variables

Mother- and father-reported GF did not account for any variance in baseline characteristics investigated. Adolescent GF was not a significant predictor of adolescent reported eating disorder psychopathology, %EBW or illness duration. However, adolescent-reported GF accounted for significant unique variance in clinician diagnosed depression [*β* = 1.92, Wald *X* ^2^(1) = 7.62, *p* = .006] and adolescent-reported depression [RCADS; *M* = 57.47, *SD* = 16.32; *β* = 14.77,Wald *X* ^2^(1) = 14.69, *p < .*001;]; clinician-diagnosed anxiety [*β* = 2.40, Wald *X* ^2^(1) = 9.49, *p* = .002] and adolescent-reported anxiety [RCADS; *M* = 50.46, *SD* = 14.72; *β* = 14.21, Wald *X* ^2^(1) = 16.13, *p <* .000]; adolescent-reported OCD symptoms [RCADS; *M* = 42.96, *SD* = 13.26; *β* = 14.21, Wald *X* ^2^(1) = 10.14, *p* = .001] and OCD impact [ChOCI-R; M = 12.93, *SD* = 12.47; *β* = 6.27, Wald *X* ^2^(1) = 4.09, *p* = .043] indicating that poorer GF was associated with greater comorbid psychiatric symptoms. Poorer adolescent GF scores were associated with AN-BP diagnosis [*β* = 1.55, Wald *X* ^2^(1) = 5.55, *p* = .018]. Better GF was associated with higher adolescent-reported self-esteem [RSES; *M* = 16.56, *SD* = 6.93; *β* = −4.55, Wald *X* ^2^(1) = 7.12, *p* = .008]; stronger attachment to mother [*β* = −5.66, Wald *X* ^2^(1) = 6.51, *p* = .011] and stronger attachment to father [*β* = −5.069, Wald *X* ^2^(1) = 4.15, *p* = .042]. GF was not a significant predictor of adolescent-reported global health (CHQ-CF87; M = 47.82, *SD* = 32.14).

### Baseline family functioning, adolescent-parent attachment and remission at session 20 and 12-month follow-up

Better adolescent-reported Problem Solving [*β* = −1.68, Wald *X* ^2^(1) = 4.759, *p* = .029; OR = 5.536, *p* = .019], Communication [*β* = −1.69, Wald *X* ^2^(1) = 4.102, *p* = .043; OR = 4.603, *p* = .032] and General Functioning [*β* = −2.69, Wald *X* ^2^(1) = 7.837, *p* = .005; OR = 12.344, *p* < .000] at baseline predicted remission at session 20, but not 12-month follow-up. Higher levels of father-reported Behavioural Control at commencement of treatment predicted remission at 12-month follow-up [*β* = 2.161, Wald *X* ^2^(1) = 4.183, *p* = .036; OR = 5.244, *p* = .022], but not at session 20. No aspects of mother-reported family functioning at baseline predicted remission status at session 20 or 12-month follow-up. Baseline adolescent attachment to mother and father did not predict remission at session 20 or 12-month follow-up.

### Changes in family functioning, adolescent-parent attachment and remission at session 20 and 12-month follow-up

Change in mother-, father- and adolescent-reported family functioning domains at session 20 or 12-month follow-up did not predict remission status at either time point. Similarly, adolescent-reported attachment quality did not predict remission status at session 20 and 12-month follow-up.

### Changes in family functioning between baseline, session 20 and 12-month follow-up

There were no main effects for time on any of the family functioning subscales for mothers or adolescents, although adolescent-reported Behavioural Control approached significance (*p* = .058). There was a main effect for time with father-reported Communication [*F*(2, 77.77) =5.32, *p* = .007, with a model pseudo-*R* ^*2*^ of .03], Affective Responsiveness [*F*(2, 75.75) =3.03, *p* = .054 with a model pseudo-*R* ^*2*^ of −.01], and General Functioning [*F*(2, 77.90) =4.23, *p* = .018, with a model pseudo-*R* ^*2*^ of .003] with each subscale more impaired at session 20 but returning to baseline levels at 12-month follow-up. Adolescent-reported attachment to mother [*F*(2, 103.20) =4.45, *p* = .014, with a model pseudo-*R* ^*2*^ of .04] and father [*F*(2, 99.75) = 4.28, *p* = .016, with a model pseudo-*R* ^*2*^ of.04] declined from baseline to session 20, and then remaining unchanged at 12-month follow-up.

## Discussion

This research investigated the impact of family functioning and adolescent-parent attachment on treatment outcomes for adolescents with high levels of AN psychopathology, hospitalised for management of medical instability prior to FBT. Firstly, the relationship between general family functioning health and AN, co-morbidity and family variables were assessed at the beginning of treatment to confirm the association with greater levels of symptomatology found in previous research [24]. Results indicated that poorer adolescent-reported general family functioning impairment predicted higher levels of comorbid psychiatric features, as well as an AN binge/purge diagnosis. This relationship between family functioning, comorbidity and AN binge/purge diagnosis from the adolescent’s perspective has been previously reported [24, 44, 48] and patients with these difficulties are vulnerable to poorer outcomes [16, 22]. Better adolescent-reported general family functioning was associated with higher adolescent self-esteem and stronger adolescent attachment with both parents, highlighting the interplay between healthy family functioning, attachment and adolescent psychosocial development [49]. These associations did not occur from the either parent’s perspective.

General family functioning in this study was not related to severity of eating disorder psychopathology, weight at admission or duration of illness, and levels of impairment were generally below the established clinical cut-offs [42]. Ciao and colleagues [24] reported baseline eating disorder pathology was associated with adolescent-reported general family functioning, and longer duration of illness associated with father-reported general family functioning [24]. However, in our group the illness duration was approximately 6 months shorter, while eating disorder psychopathology and rates of psychiatric comorbidity were substantially higher. Other FT studies [13, 23, 34, 50] report a longer duration of illness prior to treatment, indicating that our group became acutely unwell quickly, so one might hypothesise that the different finding between this study and Ciao et al. reflects the impact of greater illness duration.

Secondly, we investigated whether family functioning and attachment at the beginning of treatment, as well as changes during treatment, predicted remission. Adolescent-reported General Functioning, Communication and Problem solving at baseline predicted remission at session 20, but not follow-up. Attachment to mother or father did not predict remission at any time point. Other studies have also identified better family functioning as positively related to remission [21, 22], although direct comparisons to the current study are difficult because of different assessment measures employed. It is not surprising that adolescents who report more positive family health, better communication and problem solving, may be able to work more effectively with their parents to recover in FBT, given the emphasis on direct communication and problem solving to induce behaviour change [5]. Alternatively, adolescents who reported less family difficulty may have been more likely to remit anyway, given adolescents with better family functioning had lower rates of psychiatric comorbidity as noted above. Adolescents with poorer family functioning may be recognising pre-existing difficulties or their perspective reflects the impact of AN. Regardless, the adolescent’s perspective is crucial as the perception of poorer family functioning at baseline was a predictor of poor outcome at the end of treatment. Finding ways to address family issues early in treatment may improve outcome and this warrants further investigation. By addressing family functioning perceptions early in treatment, adolescents may experience a reduction in distress and feel more contained [51], helping to reduce the negative impact of co-morbid psychiatric problems on remission rates [22].

No elements of mother-reported family functioning at commencement of treatment were related to better outcome, however, higher levels of father-reported Behaviour Control (i.e. rules and expected behaviours) at commencement of treatment were positively related to remission status in the longer term. This seems counter intuitive as higher FAD scores usually indicate less family health. One might hypothesise that a higher score at baseline reflects the rigid control dimension (characterised by constricted range of, and low negotiation) in the McMaster model of family functioning, which forms the theory behind the FAD [41]. As the majority of adolescents did not meet remission criteria at session 20, fathers perceiving high behavioural control may have been well suited to persisting with phase 1 style parental management of AN in the longer term. This unexpected and preliminary finding requires further investigation, and highlights the difficulty in assessing family functioning in the context of a complex illness.

Thirdly, changes in family functioning and parent-adolescent relationship quality were explored. There was no overall improvement in family functioning for mothers, fathers or adolescents either during treatment or at follow-up and no significant relationship between change and remission at either session 20 or follow-up. Limited information on changes in family functioning during FBT currently exists. Previous FBT and FT studies employing different self report measures have reported improvement in family relationships and maintenance (control) from a parent perspective after 6 months of treatment [22] and positive changes in closeness for adolescent and parents after 12-months [13]. The only other FBT study to use the FAD reported improvement in communication and affective involvement at the end of treatment for mothers, fathers and adolescents [24]. The pattern in the current study was different as only changes in father-reported family functioning were significant. Interestingly, from the father’s perspective, general family health (GF), Communication and Affective responsiveness (showing affect appropriate to the situation) became more problematic by session 20, but reduced to baseline levels at 12-month follow-up. This is a new finding and possibly indicates some negative treatment impacts for fathers that dissipated after treatment. Alternatively, fathers may have had a greater awareness of family issues at session 20, but as time progressed became less concerned or adjusted to the situation and this is reflected in their response. Why this decline occurred for fathers and not mothers suggests the need to better understand treatment from different family member perspectives.

The reduction in attachment quality by session 20, which persisted at follow-up, was not expected given the relationship focus in the final stage of FBT. This finding warrants further investigation because qualitative findings with this group and other FT studies suggest improved relationship quality with parents after treatment [51, 52]. It may be that this finding reflects a negative treatment outcome, although there was no corresponding decline in adolescent perceived family functioning, which may have been expected if the decline was related to negative treatment outcome. An alternative hypothesis may be that the decline in relationship quality just reflects normal adolescent autonomy processes. A recent study with a shorter follow-up period reported a decline in attachment quality for this group, as well as for non-clinical adolescents, and no significant difference in attachment quality [53]. By the follow-up in this study patients were almost 2 years older, and older adolescents appear to report lower attachment levels with the IPPA when compared to younger teens [54]. Previous FBT studies have not investigated attachment quality and further research in this area is important because the current FBT model delays specific relationship and adolescent issues until later in treatment, and this may not be the most effective sequence of treatment.

Overall these findings suggest that family functioning is an important area to assess at the commencement of treatment. Adolescent-reported impairment at the commencement of treatment may indicate a need to deliver FBT with a more relational focus that considers additional ways to help the adolescent perceive that parental control is occurring with the right intention. Qualitative research suggests that it is important for adolescents to feel relationally connected to their family during FBT and this aids adolescents perceiving treatment as a unified family effort despite having control of eating taken away early in treatment [51]. Current published efforts to augment FBT have involved additional parent meal coaching early in treatment [55] or parent only FBT [34]. However, addressing relationship issues from the adolescent’s point of view may be another way to improve poor progress, especially given some initial evidence that systemic family therapy achieved a similar outcome to FBT in a recent trial [7]. Systemic family therapy as described by Agras et al. [7] allowed a focus on both relationship and eating issues, if the family indicated this need. Thus this model combined with FBT may be a potential augmentation that would suit families with both AN and family functioning or relationship difficulties and could be a focus for future research.

A limitation of this study was the use of self-report measures, and therefore the outcome may have differed with the inclusion of observational methods. However, both parent and adolescent reports were included; the measures were well validated and used in previous eating disorder research [24, 44]. Secondly, this study focused on a subset of those who completed the RCT protocol. While there were no significant differences between this group and those who completed the RCT, there is always the possibility additional data may have impacted the outcome. There are a number of strengths of the study. The patients had high levels of AN psychopathology, medical complications and psychiatric comorbidity representing the most unwell end of the AN spectrum and a group previously not included in family functioning research [24]. In addition, FBT was manualised and adolescent as well as parent perspectives considered at multiple time points.

## Conclusions

While changes in family functioning during FBT were not related to remission status, adolescent perceived functioning at the commencement of treatment did predict remission. In particular, adolescents who perceived better communication and problem solving were more likely to respond to FBT. Investigating ways to improve adolescent perceptions of their family at the start of treatment may provide another area where treatment can be modified to improve outcome, especially for adolescents who are experiencing high levels of psychiatric comorbidity. In addition, finding ways to assess and modify family functioning early in treatment may be of particular benefit given other studies have noted the importance of early treatment response for other variables, such as weight [56, 57]. This study confirms the importance of understanding the family context from multiple perspectives and it remains a key area of adolescent eating disorder research.

## Abbreviations

AN: Anorexia Nervosa
AN-BP: Anorexia Nervosa-Binge/Purge
AN-R: Anorexia nervosa-restrictive
CDC: Centre for disease control
ChOCI-R: Children’s obsessive compulsive inventory revised
CHQ-CF87: Child Health Questionaire-Child Form
DSMIV: Diagnostic and statistical manual of mental disorders
EBW: Estimated body weight
EDE: Eating disorder examination
FAD: Family assessment device
FBT: Family based treatment
GF: General Functioning
IPPA: Inventory of parent and peer attachment
K-SADS-PL: Schedule for affective disorders and schizophrenia for school aged children present and lifetime
RCADS: Revised child anxiety depression scale
RSES: Rosenberg self esteem scale
